# Characterization and Optimization of Intelligent Dampers Based on Bionic Principles

**DOI:** 10.3390/biomimetics11060411

**Published:** 2026-06-11

**Authors:** Niancheng Guo, Yujing Zhang, Hao Cheng, Wei Zhao, Yang Gao, Wei Li, Yanle Li

**Affiliations:** 1 School of Mechanical Engineering, Shandong University, Jinan 250061, China; guonc@sdu.edu.cn (N.G.); 202334480@mail.sdu.edu.cn (Y.Z.); chengh@mail.sdu.edu.cn (H.C.); 202590000201@sdu.edu.cn (W.Z.); 2 Key Laboratory of High Efficiency and Clean Mechanical Manufacture of Ministry of Education, Shandong University, Jinan 250061, China; 3 School of Automotive Engineering, Shandong Jiaotong University, Jinan 250357, China

**Keywords:** semi active suspension, damper model, vibration control, parameter optimization, Biomimetics

## Abstract

From the perspective of human vibration perception, reducing vibration stimuli transmitted to occupants is essential for improving ride comfort and reducing fatigue. Intelligent dampers, as key actuators in semi-active suspension systems, provide adjustable damping capabilities for vibration control. This article combines them with biomimetic control principles to study the vibration control of semi-active suspension. The effects of damper forward and inverse models, damping force ranges, and time delays on suspension performance were analyzed. The results show that a function prediction-based damper model, a damping force range below 0.2 times and above 1.4 times the passive curve, and a 10 ms delay could balance vibration reduction and economy. Particle swarm optimization is used to optimize LQR control parameters for different road grades and typical speeds. Inspired by the adaptive behavior of chameleons, graded weights are assigned according to road characteristics, with greater emphasis on comfort on Grade A and B roads and driving stability on Grade C and D roads. The results show that proper matching of damper models and parameter constraints can fully exploit the adjustable damping capability of smart dampers. These findings provide a theoretical basis for designing and optimizing semi-active suspension control strategies.

## 1. Introduction

As one of the most important components of a car, the suspension system plays a crucial role in buffering road shocks, absorbing vertical vibrations, and enhancing ride comfort [1,2,3,4]. In addition to improving ride comfort and handling stability, vehicle suspension systems have recently attracted attention as potential sources of vibration energy recovery [5]. Automotive suspensions are mainly divided into traditional passive suspensions, semi-active suspensions, and active suspensions. Traditional passive suspension springs have fixed stiffness and damping coefficients, making it difficult to meet the requirements of handling and comfort [6,7,8]. Compared with traditional passive suspension, active suspension is a more advanced suspension system [9]. However, active suspension systems have complex structures and controls, high manufacturing costs, and require continuous external energy supply due to their use of active actuation mechanisms, resulting in high energy consumption [10]. In contrast, semi-active suspension systems have both economic and reliability advantages while maintaining approximate active control performance, thus demonstrating greater potential for development in engineering applications [11]. Adjustable dampers feature continuously variable damping, allowing the suspension to transition smoothly between soft and firm settings. This enables faster control of body pitch and roll movements while reducing vibration, serving as the foundation for suspension control strategies [12,13,14,15].

Continuously adjustable dampers (CDCs) and magnetorheological dampers (MRCs) are widely used in semi-active suspension systems, and numerous researchers have conducted extensive studies on these two types of dampers. Lee et al. [13] proposed an efficient active suspension system based on CDC. When active performance is required, ride performance is controlled by the actuator, and in other cases it is controlled by CDC. Xu et al. [14] introduced damper into semi-active suspension and introduced Parallel-compound Fuzzy and PID (PcFPID) control to adjust the damping force of CDC damper, thereby improving the overall performance of suspension. Lau et al. [15] designed MR dampers for railway vehicles, tested their performance, and compared them with passive suspension systems. Sun et al. [16] developed a full-scale MR suspension system with adjustable stiffness and damping characteristics. Compared with other suspensions, it has been verified that it reduces body acceleration.

As is well known, there is a trade-off between body acceleration and dynamic tire load [17,18]. However, the driving scenarios of vehicles are complex and varied. When driving on highways, it is necessary to ensure comfort while also considering tire dynamic load and grip when driving at high speeds on certain unpaved or off-road surfaces. In existing control theories, classical control theories such as the skyhook control strategy only focus on body acceleration and cannot directly optimize suspension working space and dynamic tire load [19,20,21,22]. However, when a car is driving on rough roads, it is necessary to optimize the dynamic tire load to ensure tire ground contact force. Therefore, the skyhook control theory has limitations in its application scenarios. Linear Quadratic Regulator (LQR) control can simultaneously optimize body acceleration, suspension working space and dynamic tire load and can adjust the optimization magnitude of the three based on actual needs to achieve ideal results. Therefore, it is widely used in the field of vehicle suspension control [23,24,,25]. Gokul et al. [26] used particle swarm optimization (PSO)-based LQR control strategy to optimize the air suspension system and compared it with PID control to verify that the control strategy has good performance.

However, existing research has largely focused on improving the control algorithms themselves, and consideration of damping system engineering implementation factors remains insufficient. In actual semi-active suspension systems, factors such as the accuracy of the damper forward-reverse model, the adjustable range of damping force, and actuation delay directly affect the effectiveness of control force tracking and the results of suspension performance optimization. If these practical boundary conditions are ignored, the ideal performance achieved by the controller in simulation may be difficult to fully realize in the actual system. In addition, there are significant differences in vehicle vibration characteristics and control requirements on different road surfaces, making it difficult for fixed control weights to meet the performance demands of both smooth and rough road surfaces simultaneously. Therefore, it is necessary to conduct further research on the optimization of control parameters for different road surface conditions, based on the actual constraints of the dampers.

Inspired by the mechanisms by which living organisms perceive, respond to, and adapt to external environmental stimuli, this paper combines the adjustable damping characteristics of intelligent dampers with biomimetic optimization principles to conduct research on the optimization of semi-active suspension control parameters. On the one hand, a particle swarm optimization algorithm inspired by swarm intelligence is employed to optimize the LQR control weights, thereby reducing the subjectivity associated with traditional trial-and-error methods; on the other hand, drawing on the biomimetic concept of how chameleons adapt to environmental changes, different road surface conditions are treated as distinct external excitation environments, and the emphasis of the control objectives is adjusted according to variations in the intensity of road surface excitation.

Based on the above considerations, there are few studies that examine the impact of the damper’s own parameters (such as forward and inverse damper models and force ranges) and time delays on actual control performance. Therefore, this paper investigates the impact of practical factors—such as the forward and inverse models of dampers, the adjustment range of damping forces, and the actuation delay of dampers—on the optimization of suspension performance metrics, based on a semi-active suspension system and employing an LQR control strategy. Finally, for different road surface conditions, common driving speeds are set, and the particle swarm optimization algorithm is used to optimize the LQR parameters. This identifies the optimal control parameters under actual boundary conditions, thereby improving the optimization results for sprung mass acceleration and tire dynamic load and enhancing ride comfort. The rest of this article is organized as follows. Section 2 established the vehicle dynamics model and LQR control strategy. Section 3 analyzed the effects of forward and reverse models of dampers, damping force range, and time delay on suspension performance. Section 4 developed PSO-optimized LQR control strategy and biomimetic graded weighting scheme for different road conditions. Finally, Section 5 summarizes the main conclusions.

## 2. Semi-Active Suspension System with Adjustable Damper

### 2.1. Suspension Model Establishment

**Figure 1:**
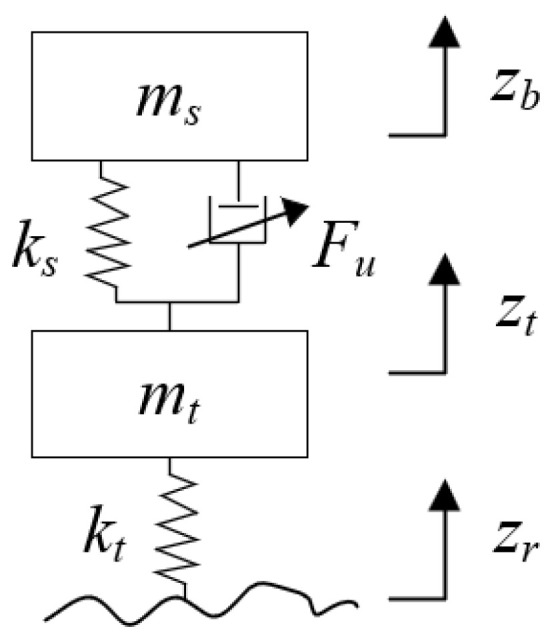
1/4 semi-active suspension model.

The quarter vehicle (1/4 vehicle), two degrees of freedom semi-active suspension model can reflect the vertical vibration of the suspension, including a spring and a damper. Variable stiffness suspension can adjust stiffness according to changes in spring-loaded mass, improving vehicle smoothness, but its manufacturing process is complex and costly. A fixed stiffness suspension can basically achieve the same effect as a variable stiffness suspension, so a fixed stiffness suspension was used in this study. The suspension model is shown in Figure 1, where $m_{s}$ is the sprung mass, the unit is kg, $m_{t}$ is the unsprung mass, the unit is kg, $k_{s}$ is the suspension stiffness, the unit is N/m, $k_{t}$ is the tire stiffness, the unit is N/m, $z_{b}$ is the sprung mass displacement, the unit is m, $z_{t}$ is the unsprung mass displacement, the unit is m, $z_{r}$ is the road displacement excitation, the unit is m, and $F_{u}$ is the adjustable damping force, the unit is N.

The suspension dynamics equation is as follows:

(1) $$\left\{\begin{array}{l} \dot{x}=Ax+Bu+Ew\\ y=Cx+Du \end{array}\right.$$

Define the system state vector as x and control the input vector as u. Where $x={[z_{b}-z_{t}\;\dot{z}}_{b\;}z_{r}-z_{t}\;{\dot{z}}_{t}]$’, its units are in the order of m, m/s, m, m/s; $u=\left[F_{u}\right].$

$$\begin{array}{l} A=\left[\left.\begin{array}{cccc} 0 & 1 & 0 & -1\\ -\frac{k_{s}}{m_{s}} & 0 & 0 & 0\\ 0 & 0 & 0 & -1\\ \frac{k_{s}}{m_{t}} & 0 & \frac{k_{t}}{m_{t}} & 0 \end{array}\right]\right.;\;E=\left[\left.\begin{array}{c} 0\\ 0\\ 1\\ 0 \end{array}\right]\right.;\;C=\left[\left.\begin{array}{cccc} \frac{k_{s}}{m_{s}} & 0 & 0 & 0\\ 1 & 0 & 0 & 0\\ 0 & 0 & 1 & 0 \end{array}\right]\right.;\\ D=\left[\left.\begin{array}{c} \frac{1}{m_{s}}\\ 0\\ 0 \end{array}\right]\right.;w=\left[{\dot{z}}_{r}\right];\;y=\left[\left.\begin{array}{c} {\ddot{z}}_{b}\\ z_{b}-z_{t}\\ z_{r}-z_{t} \end{array}\right]\right. \end{array}$$

The unit of *w* is m/s, the unit of *y* are m/s^2^, m, and m. Among them, y represents the spring mass acceleration, suspension working space, and tire dynamic deflection, respectively. In this paper, m_s_ = 2396 kg, m_t_ = 287 kg, k_s_ =108,000 N/m, and k_t_ =,1,200,000 N/m.

### 2.2. LQR Control Strategy Based on Semi-Active Suspension

Linear Quadratic Regulator (LQR) is an optimal control method. In suspension systems, LQR can simultaneously take into account body acceleration, suspension working space and dynamic tire load, and allow the suspension system to perform ideally under different operating conditions. The control objective function of LQR is as follows:

(2) $$J=\int \begin{array}{l} \infty \\ 0 \end{array}[q_{1}{\ddot{z}}_{b}^{2}+q_{2}{\left(z_{b}-z_{t}\right)}^{2}+q_{3}{\left(z_{r}-z_{t}\right)}^{2}+q_{4}u^{2}]dt$$

where *q*_1_, *q*_2_, *q*_3_, and *q*_4_ are the control objective weights of LQR. Among them, *q*_1_, *q*_2_, and *q*_3_ are the weight coefficients corresponding to the spring mass acceleration, suspension working space, and tire dynamic deformation, respectively. *q*_4_ is the weight coefficient corresponding to the shock absorber control force *F*_u_. The purpose of introducing q_4_ is to constrain the control force amplitude in the performance indicator function to avoid excessive control effects. Due to the fact that the research object of this article is a semi-active suspension system and the performance indicators do not involve the main driving force, q_4_ is usually set to 0 [19].

Combined (2)—rewrite the control objective function into a more general form:

(3) $$J=\int \begin{array}{l} 0\\ \infty \end{array}(x^{T}Qx+u^{T}Ru+2x^{T}Nu)dt$$

$Q_{1}=diag[\begin{array}{ccc} q_{1} & q_{2} & q_{3}] \end{array}$, where $Q=C^{T}Q_{1}C$, $R=D^{T}Q_{1}D+q_{4}$, $N=C^{T}Q_{1}D$.

The optimal feedback control gain matrix for the objective function is as follows:

(4) u=−Kx

where $K=R^{-1}B^{T}P$. According to Riccate, P is obtained as follows:

(5) $$PA+A^{T}P-PBR^{-1}B^{T}P+Q=0$$

According to the above formula, selecting the values of *q*_1_, *q*_2_, and *q*_3_ can achieve the ideal control effect, suppress body vibration, and improve tire grip.

### 2.3. Comparison and Analysis of Ideal Semi-Active Suspension Systems and Actual Semi-Active Suspension Systems

In actual semi-active suspension systems for vehicles, the damping force output by damper is constrained by both its structure and the characteristics of the working medium; thus, there exists a clear achievable force range. However, in idealized models, this physical limitation is usually ignored, and there are no restrictions on the upper and lower limits of the damping force. If the damping force is considered to be freely adjustable, the controller can output the optimal damping force under any road conditions, thus maximizing the balance and optimization of both body acceleration and dynamic tire load. However, when the required damping force exceeds the design limits of the damper, it is impossible to apply this amount of force in practice, thus causing a deviation between the control strategy and physical execution and losing the true reflection of the constraints on vehicle comfort and handling stability. Therefore, in the design of semi-active suspension control, it is necessary to set the maximum and minimum achievable damping forces of the damper to ensure the feasibility and safety of the theoretical optimization results in engineering practice.

To analyze the control performance of the LQR controller under different road conditions under ideal conditions, simulations were conducted under random road surface excitations of levels A, B, C, and D. The evaluation indicators include the vertical acceleration of the spring mass, suspension working space, and tire dynamic load, all of which are evaluated using Root Mean Square (RMS) values. Table 1 presents the performance comparison results of passive suspension and ideal LQR-controlled suspension under different road conditions. Among them, passive represents the passive suspension system, and ideal model represents the ideal LQR control result without considering the nonlinear characteristics of the shock absorber and actuator constraints. The results show that, compared with passive suspension, body acceleration on A-level road is optimized by 33.9%, body acceleration on B-level road is optimized by 28.5%, 15.6% optimization of body acceleration is under C-level road, and 6% optimization of body acceleration is under D-level road. The optimization effect is good.

**Figure 2:**
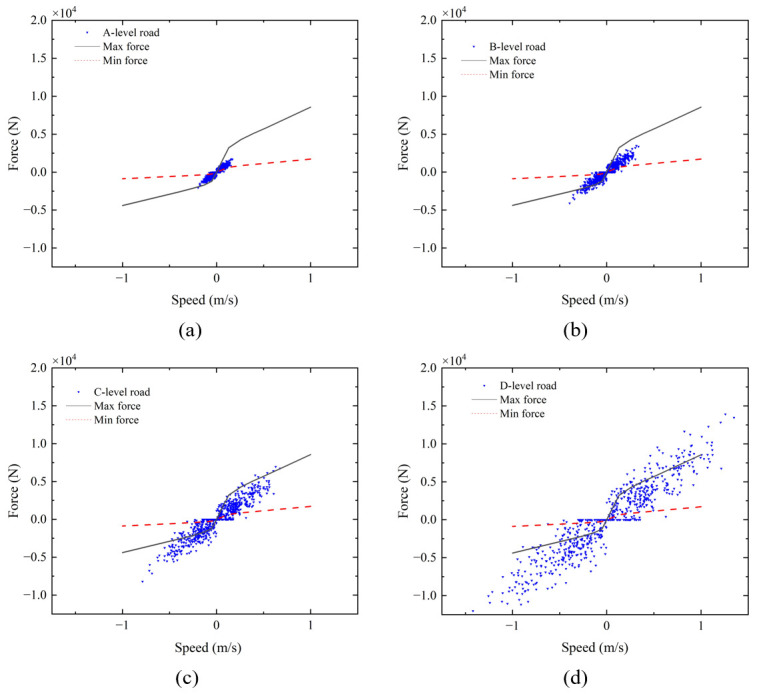
Ideal damping force curve. (**a**) A-level road; (**b**) B-level road; (**c**) C-level road; (**d**) D-level road.

Based on the established vehicle dynamics model and LQR control strategy, simulation calculations were conducted in Simulink environment. The controller outputs the target control force in real-time based on the system state feedback, which is the ideal damping force. Under ideal operating conditions, the damping forces of the damper corresponding to road surfaces of grades A, B, C, and D are shown in the scatter plot in Figure 2.

In this scenario, the damper’s output force is not constrained by actual physical conditions but instead demonstrates the damper’s optimal mechanical performance on different road surfaces. The figure also shows the output range that the damper can achieve under actual physical conditions, with the red line representing the minimum range and the black line representing the maximum range. When the vehicle is traveling on relatively smooth road surfaces (Levels A and B), lower damping force is required; therefore, the damping force exceeds the damper’s minimum range only in isolated instances. When the vehicle encounters rougher road surfaces (Levels C and D), the damping force not only exceeds the minimum range but also, at certain points, exceeds the damper’s maximum adjustable range. This overshoot phenomenon not only contradicts the physical capabilities of the actual hardware but also causes the control strategy to fail in engineering applications, making it impossible to guarantee the vehicle’s comfort and stability performance. Therefore, to enhance the engineering applicability of simulation results, the semi-active suspension control model must incorporate upper and lower limits for the dampers and restrict the damping force range within the optimization algorithm to ensure consistency between theoretical design and actual implementation.

The purpose of the damper forward-reverse model is to convert the optimal control force required by the suspension system into a control force that the damper can actually produce. When calculating the optimal suspension control force, it is necessary to consider not only the control algorithm but also the range of forces that the damper can exert at different speeds. In other words, compared to the ideal damping force, the actual damping force must be calculated by applying a saturation operation to the ideal damping force at different speed points, based on the damper’s specific performance characteristics. In addition, the operating speed range of a damper must be determined through testing. Since test speeds are necessarily limited by the capabilities of the testing equipment, the maximum test speed for dampers is typically 1–2 m/s. This test speed may not cover the maximum speed of the damper under Level D or even lower road conditions. Therefore, for damping force curves beyond the measured speed range, the inverse model should incorporate appropriate extrapolation algorithms to ensure a smooth transition or reasonable estimation of data outside the calibration range.

At the same time, in actual semi-active suspension systems, the force output of the damper does not respond instantaneously to control commands. Instead, it exhibits a certain degree of dynamic lag due to various dynamic factors, including the movement of the valve spool, the establishment of a stable flow field as fluid flows, changes in the viscosity of the magnetorheological fluid, and the running-in of the valve body seals. The actual model must account for this time delay effect in order to reflect the impact of lag in the real system on suspension control performance, thereby providing a more reliable engineering basis for the robustness analysis and optimization of control algorithms [27].

## 3. Factors Affecting Dampers

### 3.1. Impact of Damper Inverse Model

As an adjustable damping force actuator, the modeling accuracy of damper has a decisive influence on the control performance of semi-active suspension systems. An accurate model can effectively predict the mapping relationship between the output force of the damper and the control command, thereby ensuring execution accuracy. Damper models are divided into positive model and inverse model. Among them, the inverse model outputs control current to the positive model by inputting the relative speed of the suspension and the optimal damping force calculated by the controller. The work can be divided into the following two parts: the first part calculates the damping force corresponding to different currents based on the relative speed of the suspension. It is necessary to determine whether the relative speed of the suspension exceeds the test speed range of the damper and calculate the damping force using different numerical methods within and outside the range. In the second part, the control current is calculated by interpolation using the optimal damping force based on the current damping force relationship data obtained from the suspension relative speed. At the same time, the control current must be limited within the range of the damper. The control current is obtained from the inverse damper model based on the target damping force and the relative velocity of the damper. Changes in the target damping force or velocity will result in changes in the control current. During the simulation optimization process, the positive model obtains the final control force based on the current output from the inverse model and the relative speed of the suspension. Its job responsibilities can also be broken down into the following two parts: the first part is the same as the first part of the inverse model, and the second part calculates the control force by interpolating the current output from the inverse model based on the obtained current damping force correspondence data.

In the first part of the inverse model of the damper, the current method for calculating the damping force within the speed range mainly adopts polynomial interpolation, such as [28], but this method has many coefficients and the calculation is cumbersome. Therefore, this study proposes a new interpolation calculation method. When the relative suspension speed v is within the actual test speed range of the damper, the interpolated damping force is obtained by interpolating the measured data Fi(v) for each current using the relative suspension speed. Based on the set damping force and velocity fitting function, the first-order difference quotient is used to obtain the local linear relationship between the damping force and velocity:

(6) $$f\left[v_{k},v_{k+1}\right]=\frac{f_{k+1}-f_{k}}{v_{k+1}-v_{k}}$$

where $v_{k}$ is the kth velocity point, and $f_{k}$ is the damping force at velocity $v_{k}$ Further, calculate the damping force using Newton’s recursive formula:

(7) $$N_{1}(v)=f_{k}+f\left[v_{k},v_{k+1}\right]\times (v-v_{k})$$

When the relative speed of the suspension exceeds the range, based on the characteristics of the damper, this study proposes an out-of-range approximation prediction method. The excess force is predicted using the function $f=C*\sqrt[{3}]{v}$, where C is solved based on the origin and test speed boundary points. In this study, taking current conditions of 1 A and 2 A as examples, interpolation fitting and function prediction were performed on the force–velocity characteristic curves of the damper within the velocity range (|v| ≤ 1 m/s) and outside the range (|v| > 1 m/s), respectively. For data within the speed range of [–1, 1] m/s, an interpolation method was used to accurately fit the measured curve for ensuring high fitting accuracy within the region fully supported by the data. When the speed exceeds this range, due to the lack of experimental data, an extrapolation method based on empirical functions is used to predict the force–velocity relationship in the unknown segment.

**Figure 3:**
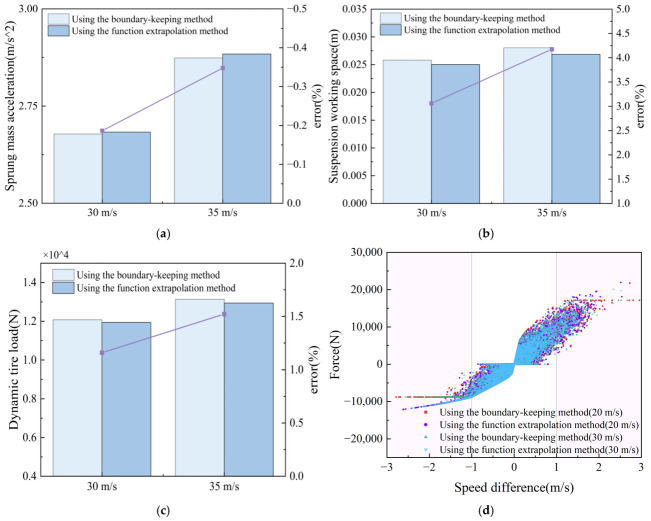
Comparison of forward and inverse models using the boundary-preserving method and the function extrapolation method. (**a**) Sprung mass acceleration; (**b**) suspension working space; (**c**) dynamic tire load; (**d**) speed difference force curve.

Set the vehicle speed to 30 m/s and 35 m/s and simulate the suspension performance index RMS value and the error between the two models using the function extrapolation method in this article and the traditional boundary preservation method, respectively, as shown in Figure 3.

In Figure 3, the performance of function method and boundary preservation method on different suspension performance indicators is not completely consistent. This indicates that the handling method of the external damping force not only affects the estimation accuracy of the damping force itself, but also changes the performance allocation relationship between smoothness, handling stability, and working stroke of the suspension system. Compared to the boundary-keeping method, the function extrapolation method places greater emphasis on providing a continuous description of the trend in out-of-boundary damping force. As evidenced by the errors, suspension working space is the most sensitive factor affecting the prediction error of out-of-boundary damping force. As vehicle speed increases, errors in all three metrics increase, indicating that at high speeds, the frequencywith which dampers enter the out-of-boundary operating region increases, causing the discrepancy between the extrapolated model and the ideal model to become more pronounced. When the velocity exceeds 1 m/s or falls below −1 m/s, the data fall within the extrapolation region of the damper function. As can also be seen in Figure 3d, the higher the velocity, the more data points fall within the extrapolation region.

**Figure 4:**
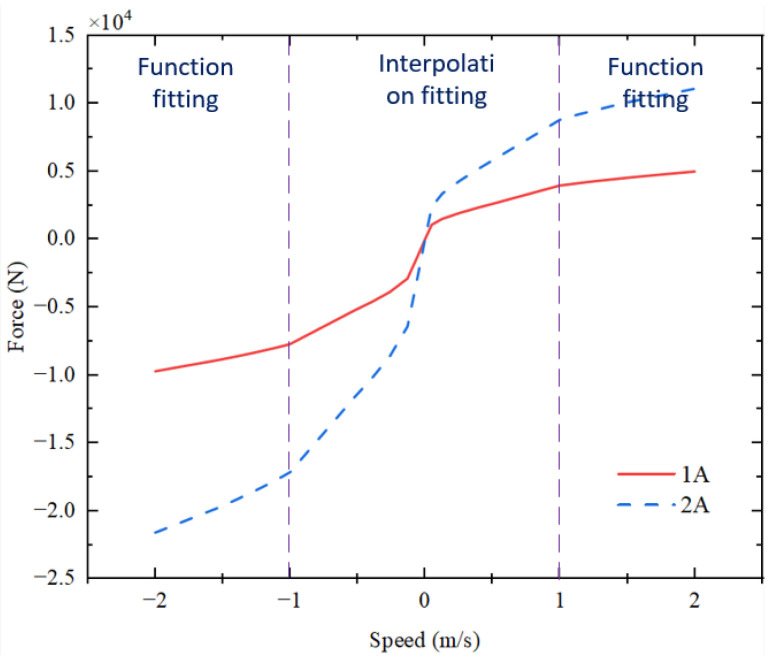
Damper damping fitting and prediction curve.

As shown in Figure 4, the transition between the fitting segment and the prediction segment is smooth, enabling accurate capture of the mechanical characteristics of the damper under conditions exceeding the speed range. Therefore, the combined fitting strategy of interpolation and function extrapolation not only improves the prediction ability of the force–velocity curve under conditions beyond the speed test range but also provides a reliable mathematical model basis for damper design and simulation analysis.

### 3.2. Study on the Range of Damper Force

The damping force range of a damper refers to the range between the minimum and maximum damping forces output by the damper. This range directly determines the controller’s ability to suppress body vibration and maintain tire grip when faced with different road excitations. A narrow damping force range may result in the damper being unable to provide sufficient damping force under certain excitations, thereby failing to ensure the effectiveness of vibration suppression. Increasing the damping force range can improve the vibration suppression effect. During the compression and rebound strokes of the damper, oil flows within the chamber, generating damping force. Due to the influence of pore size and oil viscosity, the damping force has a certain range of magnitude [29]. Therefore, it is necessary to design a reasonable force range for the damper to ensure suppression while reducing energy loss.

**Figure 5:**
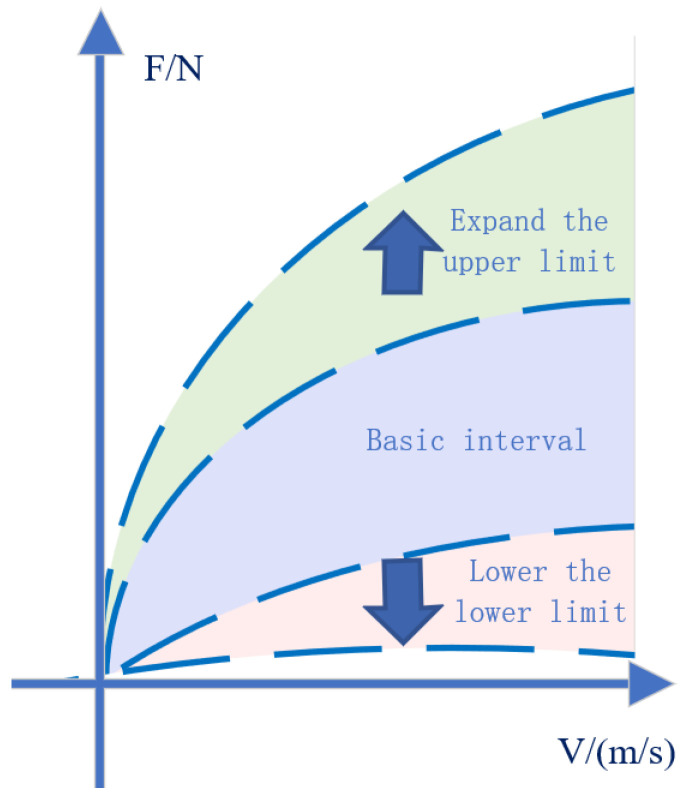
Diagram showing the upper and lower limits of damping force changes.

The force characteristic curve of the semi-active suspension damper under different current gears is obtained by multiplying the passive damper mechanical characteristic curve by the base multiplier. To simulate the opening and closing characteristics of the damper, the force increases non-linearly under different currents. To study the influence of changes in the upper and lower limits of force, multiply the passive damping force by the maximum and minimum amplification factors, as shown in Equation (8):

(8) $$f=(r_{\min}+(r_{\max}-r_{\min})*[{\begin{array}{ccc} n_{1} & \ldots & n_{i}] \end{array}}^{T})*cf$$

where r_min_ is the minimum amplification factor, r_max_ is the maximum amplification factor, i is the current gear number, ni is a monotonically increasing coefficient, and cf is the mechanical characteristic curve of the passive damper force. As shown in Figure 5, increasing the maximum multiplier raises the upper limit of the damping force, while decreasing the minimum multiplier lowers the lower limit of the damping force.

**Figure 6:**
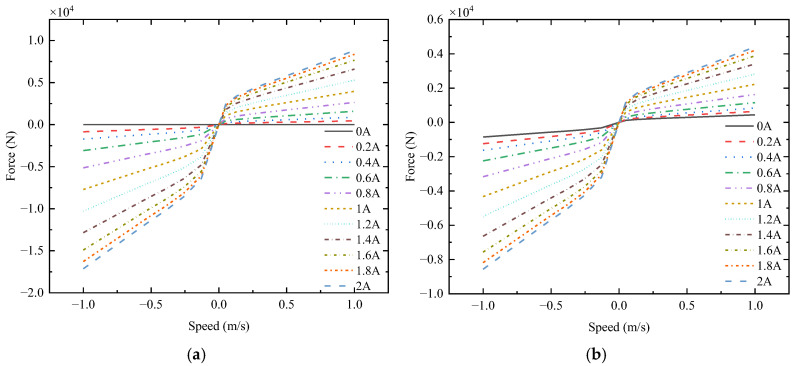
Mechanical characteristics curve of damper. (**a**) A curve with a minimum multiplier of 0 and a maximum multiplier of 2; (**b**) a curve with a minimum multiplier of 0.1 and a maximum multiplier of 1.

If the minimum factor is set to zero, the maximum factor to two, and the minimum factor to 0.1, and the maximum factor to one, the mechanical characteristic curve of the damper is obtained as shown in Figure 6. Positive velocity denotes rebound motion, and negative velocity denotes compression motion.

The portion of the damper speed greater than zero is the restoring damping force, and the portion less than zero is the compression damping force. The greater the working current, the greater the damping force provided by the damper. If the range of damping force is changed, can the performance index optimization effect be improved? Further verification will be conducted below.

**Figure 7:**
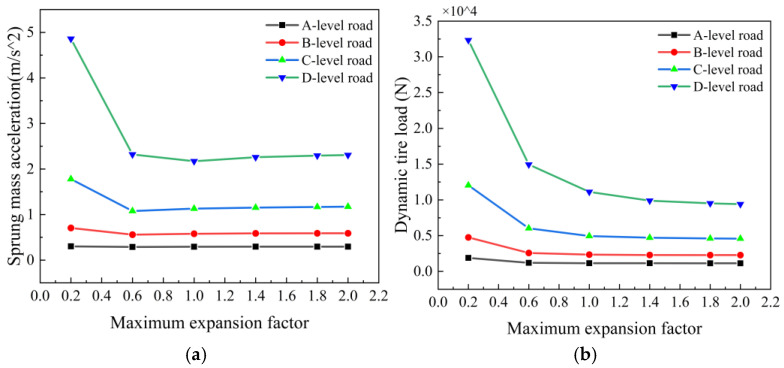
Suspension performance indicators when changing the maximum expansion factor. (**a**) Curve of sprung mass acceleration; (**b**) curve of dynamic load tire.

Based on the LQR control strategy semi-active suspension model, the minimum amplification factor of the damper damping force was set to zero, and the maximum amplification factor was changed. Simulations were performed on A-, B-, C-, and D-level road, and the RMS value values of the body acceleration, suspension working space, and dynamic tire load were recorded. The results are shown in Table 2. Plot the RMS value of the body acceleration and dynamic tire load as a line graph as shown in Figure 7. From the graph, it can be seen that as the maximum expansion factor increases, the control force increases, and the body acceleration and dynamic tire load values show an overall optimization trend. Due to the fact that the excitation intensity and frequency spectrum characteristics of C- and D-level road are more severe than those of A- and B-level road, the damper must output greater damping force to suppress the relative movement between the car body and the wheels. Therefore, increasing the upper limit of damping force can include more ideal damping force in the force range, resulting in a more significant increase in body acceleration and dynamic tire load. The rate of change between adjacent expansion factors is used as an evaluation indicator. When the rate of change in body acceleration and dynamic tire load is less than 6%, the system is considered to be stable. It follows that when the maximum expansion factor is 1.4, the body acceleration and dynamic tire load of A-, B-, C-, and D-level road are all less than 6%. Further increasing the maximum damping force limit of the dampers no longer significantly improves the acceleration of the sprung mass or reduces dynamic tire loads. At this point, the maximum damping force of the dampers can meet the maximum force requirements when the vehicle is traveling on Class A, B, C, and D road surfaces.

**Figure 8:**
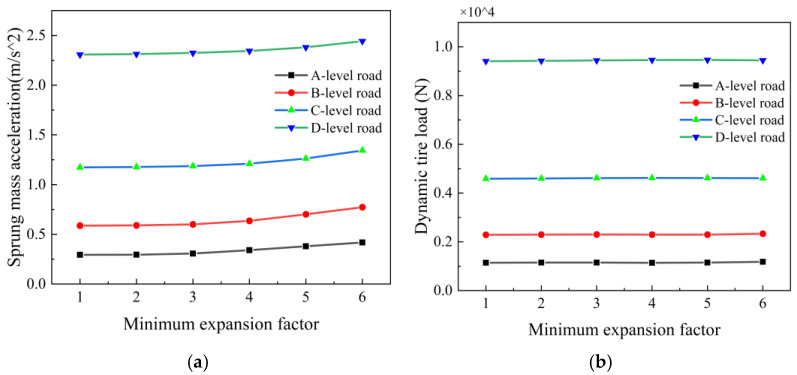
Suspension performance indicators when changing the minimum expansion factor. (**a**) Curve of sprung mass acceleration; (**b**) curve of dynamic load tire.

If the maximum expansion factor of the damper damping force is set to two, change the minimum expansion factor, simulate the A-, B-, C-, and D-level road, and record the RMS values of the body acceleration, suspension working space, and dynamic tire load, respectively. The results are shown in Table 3. Plot the RMS value of the body acceleration and dynamic tire load as a line graph as shown in Figure 8.

The Figure 8 shows that as the minimum factor increases, the overall trend of body acceleration and dynamic tire load deteriorates. This is because the increase in the expansion factor reduces the range of damping force, and the suspension system cannot obtain the required small damping due to force limitations, thus unable to adjust the vehicle’s state more accurately. Due to the good road conditions of A- and B-level road, the damper needs to output a small damping force for a longer period of time and is more affected by factor. When the minimum multiple is 0.2 times, the range of force can meet the damping force requirements of the vehicle when driving on A, B, C, and D road surfaces.

Based on the above analysis, expanding the upper limit of the force range has a greater impact on Level C and D road surfaces than on Level A and B surfaces, while expanding the lower limit of the force range has a greater impact on Level A and B surfaces than on Level C and D surfaces. This is because Level C and D road surfaces are in poorer condition, requiring greater control force to maintain vehicle stability, whereas Level A and B road surfaces are in better condition, making it more common for control force to be at lower values. In addition, an appropriate damping force range should be selected based on specific vehicle parameters and typical road conditions to balance damper performance, cost, and reliability. Considering the optimization of suspension performance metrics, this paper sets the damper range to be between 1.4 times and 0.2 times the passive curve.

**Figure 9:**
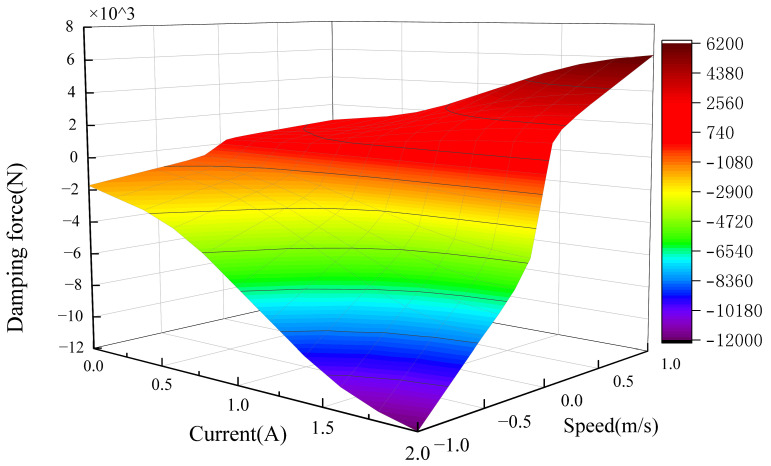
Three-dimensional electrical characteristics diagram of damper.

After defining the range of the damping force, a three-dimensional fit of the damper data against speed, current, and damping force was performed, as shown in Figure 9. Subsequently, control strategy analysis was conducted based on this damper model. From Figure 9, it can be seen that the damping force is influenced by both the piston speed and the control current. When the piston speed increases, the energy dissipation process inside the shock absorber is enhanced, and the damping force increases accordingly; when the control current increases, the adjustable damping capacity of the shock absorber is enhanced, and it can output greater damping force under the same speed conditions. Therefore, Figure 9 presents a nonlinear relationship where speed, current, and damping force increase synchronously.

### 3.3. Impact of Delay Modules

Dampers generally have a certain amount of operating delay when responding to control signals. This operating delay is mainly caused by actuator action delay and lag in the signal processing chain. The length of the delay time affects the response speed of the damper to the controller’s adjustment commands, thereby affecting the controller’s optimization of the overall performance for the suspension system and leading to reduced accuracy [30]. Therefore, in the design of semi-active suspension control strategies, the impact of actuator delay effects should be fully considered. This article uses a first-order inertia model to simulate the delay characteristics of the system.

The delay module equation is as follows:

(9) $${\dot{F}}_{d}=-\beta F_{d}+\beta u_{1}$$

where ${\dot{F}}_{d}$ is the actual damping force, u1 is the ideal damping force, and β is the cutoff frequency of the controlled damper.

In a first-order inertial system, the output response to a unit step input can be expressed as follows:

(10) $$F_{d}=1-e^{-\frac{t}{\tau }}$$

where t is the delay time, τ is the time constant τ=1/β. Due to the exponential decay term $e^{-t/\tau }$ tending to zero only at t→∞, the system output cannot precisely reach its steady-state value at any finite moment. In order to resolve the contradiction between the theoretical limit and engineering practice, this paper defines 90% as the steady-state value, that is $90/100=1-e^{-t/\tau }$. By changing, we obtain:

(11) $$t_{90}=-\tau \ln(0.1)$$

The delay module representing a certain delay time can be drawn using the above equation.

**Figure 10:**
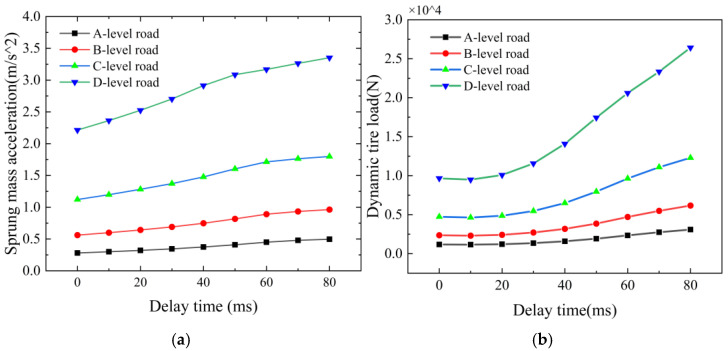
Performance indicators for suspension with delay module. (**a**) Curve of sprung mass acceleration; (**b**) curve of dynamic load tire.

A semi-active suspension model with a delay module was established based on the LQR control strategy and 20 m/s vehicle speed. Simulations were performed on A-, B-, C-, and D-level road, and the results are shown in Table 4. The curves of the body acceleration and dynamic tire load RMS values are shown in Figure 10.

The results indicate that, on road surfaces of varying grades, the LQR controller with a delay module leads to a deterioration in the control performance of both sprung mass acceleration and tire dynamic load. This is because the LQR controller makes optimal control decisions based on precise feedback of the system’s state; however, in practice, due to actuation delays in the dampers, the controller output no longer matches the system’s current state, resulting in control hysteresis. When road excitation changes, the controller cannot promptly adjust the damping force to an optimal state, leading to increased vertical vehicle vibration, worsened spring-mass acceleration, and reduced ride comfort. Delays in adjusting the contact force between the wheels and the road surface cause tire dynamic loads to worsen and reduce tire traction. However, the impact of delay varies across different road surface grades; therefore, hardware delay should be controlled within a reasonable range based on actual parameters and expected objectives. As shown in Figure 10, the impact of delay on sprung mass acceleration and tire dynamic load varies. Delays of less than 20 ms have a minor effect on tire dynamic load but a significant effect on spring-mass acceleration. Furthermore, the impact of delay differs across road surface grades, with lower-grade surfaces being more affected by delay than higher-grade surfaces.

Different dampers exhibit varying response times. The response time of the MRD damper can be controlled within 10 ms; under the control parameters described in this paper, when the response time is 10 ms, the deterioration in spring-mass acceleration on different road surface grades is within 7%. The response time of the CDC damper may reach 40 ms; under this response time, the deterioration in spring-mass acceleration on different road surface grades is 34%. The MRD damper offers certain advantages in terms of delay characteristics but is relatively expensive; while the CDC damper is more cost-effective, it has a relatively longer response delay. Therefore, in engineering applications, it is necessary to balance control performance, controller energy consumption, and control costs to select the appropriate damper type. In the subsequent research of this paper, to prioritize control performance, the delay time is set to 10 ms.

Compared with previous studies that mainly focused on suppressing suspension vibration by optimizing damping characteristics, this study also considered practical implementation factors, including damper forward and reverse models, damping force limitations, and actuator delays. The results indicate that these factors have a significant impact on achievable control performance and should be considered in controller design and parameter optimization processes.

## 4. Particle Swarm Optimization LQR

### 4.1. Optimization Objectives and Parameters of Particle Swarm Controller Based on Chameleon Multi Environment Adaptation Mechanism

The PSO algorithm is a stochastic optimization algorithm that simulates swarm. Its core idea is to maintain a particle swarm in the solution space, with each particle representing a candidate solution, and to update the position along the current velocity direction. At the same time, particles can dynamically adjust their search strategies based on information about their own historical optimal positions and global (or local) optimal positions, thereby achieving a balance between global and local searches [31]. Traditional methods rely on experience to select LQR parameters and cannot obtain the optimal solution. This study uses the PSO to find the optimal LQR weights [32,33].

**Figure 11:**
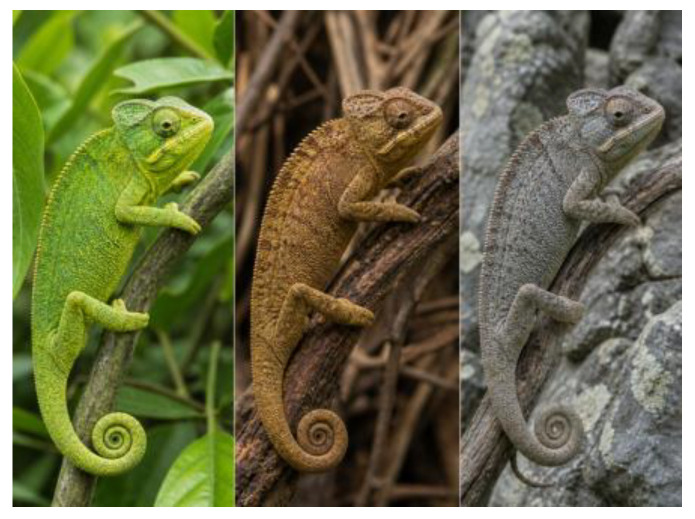
The skin color of a chameleon changes with its environment.

In the animal world, chameleons are known for their remarkable ability to adapt to their environment. They can dynamically change the color of their skin in response to external environmental conditions and their own physiological state, enabling them to adapt to their surroundings, communicate, and regulate their physiological state, such as Figure 11. This regulatory process embodies the biomimetic concept of organisms actively adjusting their response strategies to environmental changes. Drawing on the biological characteristics of chameleons that can adaptively adjust according to environmental changes, different performance index weight coefficients are designed for different levels of road surfaces and their corresponding typical vehicle speeds, so that the optimization objectives can be dynamically adjusted with changes in the external environment. On this basis, the standard PSO algorithm is used to optimize and solve the parameters of the LQR controller, in order to obtain control parameters that match the current road conditions.

The PSO parameters are *q*_1_, *q*_2_, and *q*_3_. Under A- and B-level road conditions, due to the good road conditions, the probability of tire lift-off is low, and the controller prioritizes the optimization effect of body acceleration. On C- and D-level road surfaces, due to poor road conditions, in order to ensure tire grip, the controller needs to balance body acceleration and dynamic tire load, so the weight of the dynamic tire load increases. Based on the above biomimetic adaptive control approach, the PSO weights are designed as shown in Table 5.

Since the suspension travel only needs to ensure that it does not exceed the limit, during the optimization process, the suspension working space is not set as a control objective, and only the body acceleration and dynamic tire load are considered [34]. The control objectives are set as follows:

(12) $$f(x)=\min(\sqrt{{\left[a1(\frac{ar_{s}}{ar_{p}})\right]}^{2}+{\left[a2(\frac{dr_{s}}{dr_{p}})\right]}^{2}})$$

where $ar_{s}$ is the RMS value of the body acceleration, $ar_{p}$ is the RMS value of the passive suspension body acceleration, $dr_{s}$ is the RMS value of the dynamic tire load, and $dr_{p}$ is the RMS value of the passive suspension dynamic tire load.

**Figure 12:**
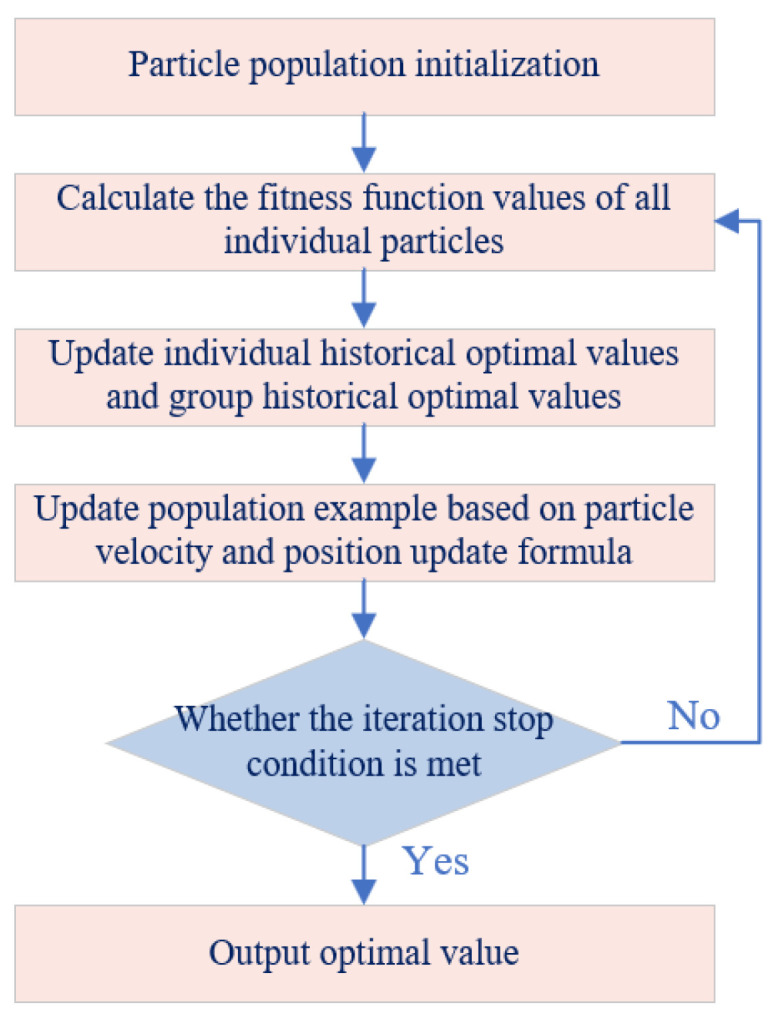
PSO flowchart.

Population size = 30, maximum iteration number = 50, inertia weight = 0.8, and learning factors c1 = c2 = 2. The PSO flowchart is shown in Figure 12.

### 4.2. Controller Parameter Optimization

Due to different road surface grades, vehicle speeds vary, and the optimal LQR parameters of PSO differ at different vehicle speeds. Therefore, based on the average driving habits of different drivers, the following different driving speeds are set for different road levels: 100 km/h on A-level road surfaces, 70 km/h on B-level road surfaces, 50 km/h on C-level road surfaces, and 30 km/h on D-level road surfaces. Based on the conclusions in Section 3, when the maximum force expansion factor is 1.4 and the minimum expansion factor is 0.2, the change rate of the adjacent factor does not exceed 6%, and the system is in a stable state. Therefore, this factor is used in the parameter optimization research model. When the damper delay time is 10 ms, there is little effect on the suspension performance indicators, and this value is within the engineering implementation range, so this value is used. The optimized results are shown in Table 6.

**Figure 13:**
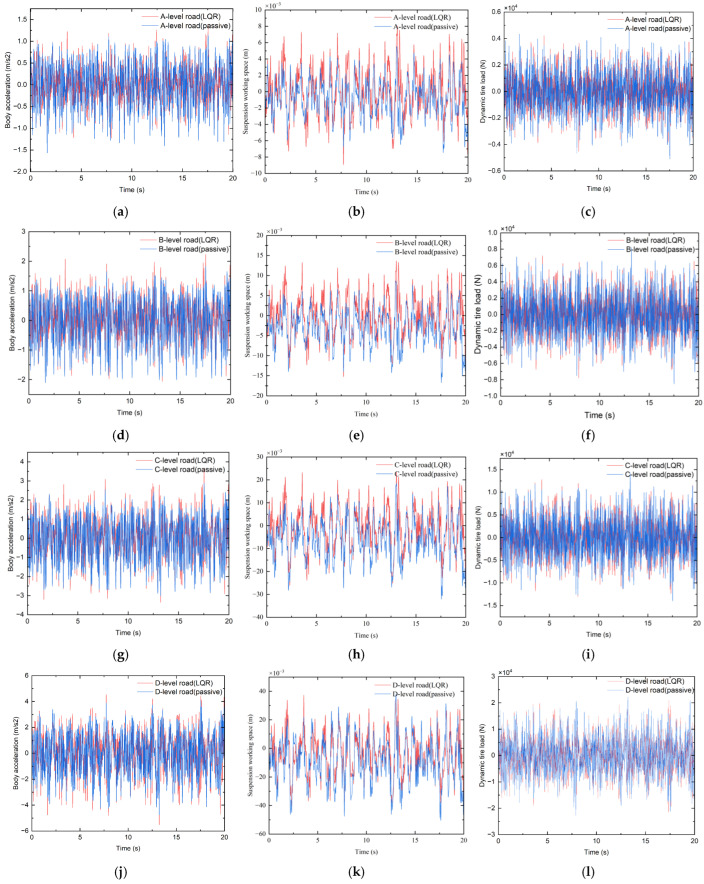
(**a**–**l**) Time domain diagram of body acceleration, suspension working space, and dynamic tire load.

Compared to passive suspensions, on Level A road surfaces, the sprung mass acceleration was optimized by 24.1% and the dynamic tire load by 6.1%; although the dynamic suspension deflection worsened, it did not exceed the limit range; on Level B road surfaces, the sprung mass acceleration was optimized by 20.8% and the dynamic tire load by 5%. On Class C road surfaces, the sprung mass acceleration was optimized by 10.3%, and the tire dynamic load by 5.1%; on Class D road surfaces, the sprung mass acceleration was optimized by 7%, and the tire dynamic load by 4.8%. Therefore, the LQR control algorithm using particle swarm optimization can meet performance metrics under different road surface classes. The above conclusions reflect the optimization results for the RMS value; the optimization results in the time domain are shown in Figure 13.

From Figure 13, it can be seen that compared to passive suspension, the optimized acceleration response peak is significantly reduced, and the vibration attenuation process is smoother, indicating that the controller can suppress vehicle body vibration caused by road excitation faster. At the same time, under different levels of road conditions, the response curve maintains good stability and there is no significant oscillation amplification phenomenon.

## 5. Conclusions

This paper investigates the effects of damper modeling, damping force constraints, and actuator delays on semi-active suspension control performance. To improve suspension adaptability under different road conditions, a biomimetic PSO-optimized LQR control strategy is proposed and validated through simulations. The main conclusions are as follows:

(1) Under ideal conditions, there are no restrictions on the control force of the damper, so the force can reach its optimal state, greatly improving the performance indicators of the suspension. However, this scenario does not reflect real-world engineering conditions. In practice, factors such as the damper’s speed range, force range, and response lag all affect the control performance and must be fully taken into account during analysis and optimization.
(2) A damper modeling method based on interpolation and function prediction was developed to establish accurate forward and inverse damper models. The results show that wider damping force ranges generally improve suspension performance, with the upper damping limit playing a greater role on Grade C and D roads and the lower damping limit being more influential on Grade A and B roads. Furthermore, actuator delay has a greater impact on smoother roads, indicating that practical implementation factors should be considered in semi-active suspension optimization.
(3) By applying PSO to optimize LQR control parameters for common vehicle speeds on different road surfaces and designing optimization weights based on control objectives, both the sprung mass acceleration and tire dynamic load have been significantly improved.

## Figures and Tables

**Table 1:** Suspension indicators under ideal conditions.

Road Surface Grade	Suspension Performance Indicators	Passive	Ideal Model
	Sprung mass acceleration (m/s^2^)	0.4245	0.2808
A-level road	Suspension working space (m)	0.0019	0.0025
	Dynamic tire load (N)	1188	1181
	Sprung mass acceleration (m/s^2^)	0.7858	0.5616
B-level road	Suspension working space (m)	0.0054	0.0049
	Dynamic tire load (N)	2371	2361
	Sprung mass acceleration (m/s^2^)	1.3310	1.1230
C-level road	Suspension working space (m)	0.0127	0.0098
	Dynamic tire load (N)	4873	4722
	Sprung mass acceleration (m/s^2^)	2.3890	2.2460
D-level road	Suspension working space (m)	0.0293	0.0196
	Dynamic tire load (N)	10,760	9444

**Table 2:** Suspension performance indicators at different maximum expansion factors.

Road Surface Grade	Suspension Performance Indicators	0.2 Times	0.6 Times	1 Times	1.4 Times	1.8 Times	2 Times
	Sprung mass acceleration (m/s^2^)	0.2987	0.2768	0.2810	0.2810	0.2809	0.2809
A-level road	Suspension working space (m)	0.0053	0.0027	0.0025	0.0025	0.0025	0.0025
	Dynamic tire load (N)	1901	1230	1187	1184	1183	1183
	Sprung mass acceleration (m/s^2^)	0.699	0.5428	0.555	0.5612	0.5618	0.5618
B-level road	Suspension working space (m)	0.0135	0.0063	0.0052	0.0050	0.0049	0.0049
	Dynamic tire load (N)	4766	2629	2419	2371	2366	2365
	Sprung mass acceleration (m/s^2^)	1.7760	1.0590	1.0900	1.1060	1.1190	1.1220
C-level road	Suspension working space (m)	0.0352	0.0158	0.0118	0.0104	0.0099	0.0099
	Dynamic tire load (N)	12,040	6067	5061	4844	4753	4736
	Sprung mass acceleration (m/s^2^)	4.8790	2.2990	2.1240	2.1800	2.2030	2.2140
D-level road	Suspension working space (m)	0.0978	0.0409	0.0284	0.0237	0.0213	0.0204
	Dynamic tire load (N)	32,380	15,010	11,220	10,120	9773	9406

**Table 3:** Suspension performance indicators at different minimum expansion factors.

Road Surface Grade	Suspension Performance Indicators	0 Times	0.2 Times	0.4 Times	0.6 Times	0.8 Times	1.2 Times
	Sprung mass acceleration (m/s^2^)	0.2810	0.2835	0.2999	0.3395	0.3797	0.4537
A-level road	Suspension working space (m)	0.0025	0.0025	0.0025	0.0025	0.0022	0.0018
	Dynamic tire load (N)	1184	1185	1172	1151	1153	1225
	Sprung mass acceleration (m/s^2^)	0.5612	0.5654	0.5808	0.6264	0.6981	0.8407
B-level road	Suspension working space (m)	0.0050	0.0050	0.0051	0.0052	0.0053	0.0052
	Dynamic tire load (N)	2371	2374	2365	2333	2314	2395
	Sprung mass acceleration (m/s^2^)	1.1060	1.1130	1.1260	1.1650	1.2310	1.4380
C-level road	Suspension working space (m)	0.0104	0.0105	0.0106	0.0109	0.0112	0.0118
	Dynamic tire load (N)	4844	4847	4847	4823	4771	4695
	Sprung mass acceleration (m/s^2^)	2.1800	2.1890	2.2070	2.2380	2.2900	2.4920
D-level road	Suspension working space (m)	0.0237	0.0235	0.0240	0.0244	0.0248	0.0257
	Dynamic tire load (N)	10,120	10,130	10,140	10,130	10,090	9875

**Table 4:** Effect of delay module on suspension performance indicators.

Road Surface Grade	Suspension Performance Indicators	No Delay	Delay 10 ms	Delay 20 ms	Delay 40 ms	Delay 60 ms	Delay 80 ms
	Sprung mass acceleration (m/s^2^)	0.2809	0.3000	0.3215	0.3743	0.4508	0.4964
A-level road	Suspension working space (m)	0.0024	0.0024	0.0025	0.0026	0.0030	0.0034
	Dynamic tire load (N)	1183	1156	1212	1591	2346	3090
	Sprung mass acceleration (m/s^2^)	0.5618	0.6000	0.6429	0.7483	0.8911	0.9628
B-level road	Suspension working space (m)	0.0049	0.0049	0.0049	0.0052	0.0062	0.0079
	Dynamic tire load (N)	2365	2312	2423	3183	4714	6170
	Sprung mass acceleration (m/s^2^)	1.1220	1.1980	1.2820	1.4770	1.7140	1.7990
C-level road	Suspension working space (m)	0.0099	0.0098	0.0099	0.0112	0.0162	0.0205
	Dynamic tire load (N)	4736	4633	4868	6497	9642	12,300
	Sprung mass acceleration (m/s^2^)	2.2140	2.3630	2.5250	2.9150	3.1680	3.3530
D-level road	Suspension working space (m)	0.0207	0.0209	0.0220	0.0294	0.0382	0.0444
	Dynamic tire load (N)	9665	9488	10,090	14,090	20,600	26,420

**Table 5:** Weighting of different road levels.

Road Surface Grade	Body Acceleration Weight (a1)	Dynamic Tire Load Weight (a2)
A-level road	0.8	0.2
B-level road	0.8	0.2
C-level road	0.5	0.5
D-level road	0.5	0.5

**Table 6:** Particle swarm optimization RMS results.

Road Surface Grade	Suspension Performance Indicators	Passive	Optimized LQR	Road Surface Grade	Suspension Performance Indicators	Passive	Optimized LQR
	Sprung mass acceleration (m/s^2^)	0.4909	0.3724		Sprung mass acceleration (m/s^2^)	1.1650	1.045
A-level road	Suspension working space (m)	0.0024	0.0028	C-level road	Suspension working space (m)	0.0104	0.0092
	Dynamic tire load (N)	1393	1308		Dynamic tire load (N)	4003	3799
	Sprung mass acceleration (m/s^2^)	0.7772	0.6153		Sprung mass acceleration (m/s^2^)	1.6450	1.53
B-level road	Suspension working space (m)	0.0053	0.0049	D-level road	Suspension working space (m)	0.0177	0.0147
	Dynamic tire load (N)	2339	2221		Dynamic tire load (N)	6531	6217

## Data Availability

The original contributions presented in this study are included in the article. Further inquiries can be directed to the corresponding authors.
